# Effect of *Uncaria rhynchophylla* against Thioacetamide-Induced Acute Liver Injury in Rat

**DOI:** 10.1155/2021/5581816

**Published:** 2021-09-13

**Authors:** Mi-Rae Shin, Min Ju Kim, Jin A Lee, Seong-Soo Roh

**Affiliations:** Department of Herbology, Korean Medicine of College, Daegu Haany University, Deagu 42158, Republic of Korea

## Abstract

Both oxidative stress (OS) and inflammation are two fundamental pathological processes of acute liver injury (ALI). The current work is to investigate the effect and possible mechanism of *Uncaria rhynchophylla* (UR) on thioacetamide- (TAA-) induced ALI in rats. UR (100 and 200 mg/kg) was orally administrated with TAA (200 mg/kg of bodyweight, intraperitoneal injection) for 3 consecutive days. ALI was confirmed using histological examination and the factors associated with OS and liver function activity measured in serum. Moreover, expressions of inflammation and collagen-related proteins were measured by the Western blot analysis. Myeloperoxidase (MPO), which mediates OS in the ALI control group, was manifested by a significant rise compared with the normal group. UR significantly reduced AST, ALT, and ammonia levels in serum. The nuclear factor-*κ*B (NF-*κ*B) activation induced by TAA led to increase both inflammatory mediators and cytokines. Whereas, UR administration remarkably suppressed such an overexpression. UR supplementation improved matrix metalloproteinases (MMPs) such as MMP-1, -2, and -8. In contrast, tissue inhibitors of metalloproteinases- (TIMP-) 1 level increased significantly by UR treatment. In addition, the histopathological analysis showed that the liver tissue lesions were improved obviously by UR treatment. UR may ameliorate the effects of TAA-induced ALI in rats by suppressing both OS through MPO activation and proinflammatory factors through NF-*κ*B activation. In conclusion, UR exhibited a potent hepatoprotective effect on ALI through the suppression of OS.

## 1. Introduction

The liver is considered as a critical hub in myriad physiological and pathophysiological processes. The liver is composed of several cell types including hepatocytes, Kupffer cells, stellate cells, biliary epithelial cells, and liver sinusoidal endothelial cells. Each of these cell types possesses special functions and cooperatively regulates hepatic function, such as the balance of metabolism [1]. Acute liver injury (ALI) has been reported to cause by various reasons, which can induce inflammation and necrosis of liver cells [2]. Thioacetamide (TAA), which is a classic hepatotoxin, causes oxidative stress (OS), membrane damage, and accumulation of lipid droplets in the hepatocyte cytoplasm to aggravate inflammation and liver injury [3]. Herein, hyperammonemia, which results from a pathological imbalance between production of ammonium and its elimination, plays a crucial role in the pathogenesis of ALI [4]. Under normal condition, ammonia present in the gut is metabolized in the liver into urea. In damaged livers, excess ammonia contributes to the development of OS and inflammation [5, 6]. The intimate correlation between inflammation and OS in the course of ALI is indisputable. A series of inflammatory response is mediated by the activation of NF-*κ*B, which plays as an important inducer of inflammation and regulates inflammatory response [7, 8]. In addition, myeloperoxidase (MPO), a heme-containing peroxidase, is highly expressed in diverse inflammatory cells, and its increase in serum was associated with the severity of liver injury [9].

The liver has a marvelous ability, both self-repair and regeneration after injury. Liver regeneration linked to the complex extracellular matrix- (ECM-) related pathways. While the normal degradation of ECM components is an important role of tissue repair and remodeling, irregular ECM turnover leads to a variety of liver diseases. Herein, ECM sustainedly undergoes remodeling mediated by matrix metalloproteinases (MMPs), which are the primary enzymes [10]. A recent study reported the distribution of MMP-1, MMP-2, and MMP-8 is related to inflammatory disease. Moreover, these factors' regulation is considered important to maintain the balance between MMPs and their tissue inhibitors (TIMPs) for tissue homeostasis [11].

*Uncaria rhynchophylla* (UR), which is known as “Gou Teng,” is one of the major components included in traditional Chinese medicine (TCM). It has been used to extinguish wind, arrest convulsions, clear heat, and pacify the liver [12]. Previous studies have shown that UR might possess vasodilation-mediating active compounds, such as indole alkaloids [13] and have a meaningful inhibitory effect on the cardiovascular system [14]. In addition, Wang et al. showed the protective effect by the alkaloid rhynchophylline on neuronal damage [15].

The purpose of this study was to investigate the hepatoprotective of UR and elucidate its underlying molecular mechanisms against ALI induced by TAA. In our experiments, UR was found to substantially inhibit the activation of MPO and also downregulate the expressions of several proinflammatory factors through the inhibition of NF-*κ*B.

## 2. Materials and Methods

### 2.1. Materials

The positive drugs silymarin, thioacetamide, and phenyl methyl sulfonyl fluoride (PMSF) were purchased from Sigma-Aldrich (St. Louis, MO, USA). The protease inhibitor mixture solution and ethylene diamine tetra acetic acid (EDTA) were purchased from Wako Pure Chemical Industries, Ltd. (Osaka, Japan). Sodium carbonate was purchased from Daejung Chemicals and Metals Co., Ltd. (Siheung, Korea). Sodium hydroxide was purchased from OCI Company Ltd. (Seoul, Korea). Phosphoric acid was purchased from Duksan Company (Ansan, Korea). The pierce BCA protein assay kit was purchased from Thermo Fisher Scientific (Waltham, MA, USA). ECL Western blotting detection reagents and pure nitrocellulose membranes were purchased from GE Healthcare (Chicago, IL, USA). Rabbit polyclonal antibodies against hemeoxygenase-1 (HO-1); goat polyclonal antibodies against tumor necrosis factor-alpha (TNF-*α*), and interleukin-1*β* (IL-1*β*); mouse monoclonal antibodies against inhibitor of nuclear factor kappa B alpha (I*κ*B*α*), nuclear factor erythroid 2-related factor 2 (Nrf2), superoxide dismutase (SOD), catalase, glutathione peroxidase-1/2 (GPx-1/2), nuclear factor-kappa B p65 (NF-*κ*Bp65), and tissue inhibitors of metalloproteinases (TIMP)-1; and mouse polyclonal antibodies against phosphoinhibitor of nuclear factor kappa B alpha (p-I*κ*B*α*), cycloxygenase-2 (COX-2), matrix metallopeptidase- (MMP-) 1, MMP-2, MMP-8, histone, and *β*-actin were purchased from Santa Cruz Biotechnology, Inc. (Dallas, TX, USA). Goat anti-rabbit, rabbit anti-goat, and goat anti-mouse immunoglobulin *G* (IgG) horseradish peroxidase- (HRP-) conjugated secondary antibodies were purchased from GeneTex, Inc. (Irvine, LA, USA).

### 2.2. Preparation of the Plant Material

*Uncaria rhynchophylla* was purchased from Ominherb Co. (Youngcheon, Korea). Each dried *Uncaria rhynchophylla* (100 g) was boiled with distilled water (1000 mL) at room temperature for 2 h, and the solvent was evaporated in vacuo to give an extract with a yield of 2.20% by weight (*Uncaria rhynchophylla*). The prepared powers were kept at −80°C until use in animal experiments.

### 2.3. Experimental Animals and Treatment

The animal experiments were performed according to “the Guidelines for Animal Experiment” approved by Ethics Committee of the Daegu Haany University (Approval No. DHU2020-081). 6-week-old male Sprague-Dawley rats (B. W. 180–200 g) were purchased from Daehan Biolink (Eumseong, Korea) and used for experiments after being adapted to the environment for 1 week. Environmental conditions were set to 12 h light/dark cycle, controlled humidity (50 ± 5%), and temperature (22 ± 2°C). After 1 week adaptation, a total of thirty-five rats were randomly divided into 5 groups as follows (*n* = 7);

  Normal: normal group
  Control: distilled water administered and TAA-induced ALI rats
  Silymarin: silymarin 100 mg/kg bodyweight administered and TAA-induced ALI rats
  UR100: UR 100 mg/kg bodyweight administered and TAA-induced ALI rats
  UR200: UR 200 mg/kg bodyweight administered and TAA-induced ALI rats

All rats were weighed once a day at a certain time, and TAA (200 mg/kg/day dissolved in distilled water) was intraperitoneally administered to all groups except for the normal group every day for 3 days with drug treatment. Drugs such as silymarin and UR administered orally 1 h 30 min prior to TAA treatment. On the day of the end of the experiment, blood was collected from the abdominal vein after anesthesia, centrifuged at 4000 rpm for 10 min (at 4°C), and then stored in −80°C freezer for biochemical estimation and ELISA analysis. Liver tissues were immediately excised and stored at −80°C.

### 2.4. DPPH and ABTS Radical Scavenging Activities

Total phenolic and flavonoid contents of *Uncaria rhynchophylla*. We followed the methods of Shin et al. [16].

### 2.5. Measurement of AST and ALT Levels in Serum

Hepatic functional parameters aspartate aminotransferase (AST) and alanine aminotransferase (ALT) assay were measured with a microplate fluorescence reader using a commercial kit (Transaminase CII-Test from Wako Pure Chemical Industries Ltd., Osaka, Japan).

### 2.6. Measurement of MPO Level in Serum

MPO was measured by the MPO colorimetric activity assay kit of BioVision, Inc. (Milpitas, CA, USA) according to the manufacturer's instructions.

### 2.7. Measurement of Ammonia Level in Serum

Serum was collected from each group as above. The levels of ammonia were measured by enzyme-linked immunosorbent assay (ELISA) kits (Abcam, Cambridge, UK) according to the manufacturer's instructions.

### 2.8. Preparation of Cytosol and Nuclear Fractions

The extraction of protein was performed according to the method of Komatsu with modifications [17]. For cytosol fractions, liver tissues were homogenized with 250 mL ice cold lysis buffer A containing 10 mM HEPES (pH 7.8), 10 mM KCl, 2 mM MgCl_2_, 1 mM DTT, 0.1 mM EDTA, 0.1 mM PMSF, and 1250 *μ*L protease inhibitor mixture solution. The tissue homogenates incubated (for 20 min at 4°C), and then 10% NP-40 was mixed well. After centrifugation (13,400 × g for 2 min at 4°C) using Eppendorf 5415R (Hamburg, Germany), the supernatant (cytosol fractions) was separated in new Eppendorf tubes. The pellets were washed twice by the lysis buffer, and the supernatant was discarded. After that, the pellets were suspended with 20 mL ice cold lysis buffer C containing 300 mM NaCl, 50 mM HEPES (pH 7.8), 50 mM KCl, 1 mM DTT, 0.1 mM PMSF, 0.1 mM EDTA, 1% (v/v) glycerol, and 100 *μ*L protease inhibitor mixture solution suspended and incubated (for 30 min at 4°C). After centrifugation (13,400 × g for 10 min at 4°C), the supernatant (nuclear fractions) was collected new Eppendorf tubes. Both cytosol and nuclear fractions were stored at −80°C before the analysis.

### 2.9. Immunoblotting Analyses

For the estimation of NF-*κ*Bp65, Nrf2, and histone, 12 *μ*g of protein from each nuclear fraction was electrophoresed through 10% sodium dodecylsulfate polyacrylamide gel (SDS-PAGE). Separated proteins were transferred to a nitrocellulose membrane, blocked with 5% (w/v) skim milk solution for 1 h, and then incubated with primary antibodies (NF-*κ*Bp65 and histone) overnight at 4°C. After the blots were washed, they were incubated with anti-rabbit or anti-mouse IgG HRP-conjugated secondary antibody for 1 h at room temperature. In addition, 12 *μ*g proteins of each cytosol fraction of Keap1, HO-1, SOD, catalase, GPx-1/2, I*κ*B*α*, p-I*κ*B*α*, COX-2, TNF-*α*, IL-1*β*, MMP-1, -2, -8, TIMP-1, and *β*-actin were electrophoresed through 8–15% SDS-PAGE. Each antigen-antibody complex was visualized using ECL Western blotting detection reagents and detected by chemiluminescence with Sensi-Q 2000 Chemidoc (Lugen Sci Co., Ltd., Gyeonggi-do, Korea). Band densities were measured using ATTO Densitograph software (ATTO Corporation, Tokyo, Japan) and quantified as the ratio to histone or *β*-actin. The protein levels of the groups are expressed relative to those of the normal rat (represented as 1).

### 2.10. Histological Examination

Histological examination microscopic was performed to evaluate the separated liver tissue. The separated liver tissue was fixed through a 10% neutral-buffered formalin and embedding in paraffin and cut into 2 *μ*m sections and stained using hematoxylin & eosin (H&E) for microscopic evaluation. The stained slices were observed under an optical microscope and then analyzed using the I-Solution Lite software program (Innerview Co., Korea).

### 2.11. Statistical Analysis

Data are presented as the mean ± SEM. Statistical comparisons were analyzed by one-way ANOVA tests followed by the least significant difference (LSD) test using SPSS (version 25.0, IBM, Armonk, NY, USA). Values of *p* < 0.05 were considered significant.

## 3. Results

### 3.1. In Vitro Antioxidant Properties of UR

**Figure 1:**
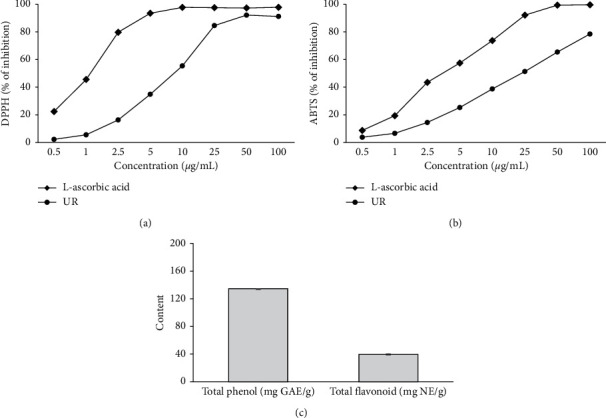
In vitro antioxidant properties of UR. (a) DPPH free radical scavenging activity. (b) ABTS free radical scavenging activity. (c) Total phenolic and flavonoid contents. Each experiment was run in triplicate. The L-ascorbic acid was used as the standard sample.

This study was performed to evaluate the antioxidant activity of UR through DPPH and ABTS radical scavenging activities. As shown in Figures 1(a) and 1(b), the DPPH IC50 value of UR was found 8.70 ± 0.47 *μ*g/mL and ABTS IC_50_ value of UR was 23.52 ± 0.91 *μ*g/mL. Moreover, total phenolic and flavonoid contents were also performed as another antioxidant agent. Total phenolic content was 134.54 ± 0.03 mg gallic acid equivalents (GAE)/g of UR extract. The flavonoid content was 39.63 ± 0.59 mg naringin equivalent (NE)/g of UR extract, as shown in Figure 1(c). Accordingly, these potent antioxidant capacities showed that UR could prevent oxidative stress induced by ALI.

### 3.2. Effect of UR on Liver Weight, Bodyweight, and Relative Liver Weight

As given in Table 1, TAA treatment significantly reduced the bodyweight (*p* < 0.001) and significantly elevated the liver weight (*p* < 0.001), while UR treatment has no statistically significant effect on the bodyweight as well as the liver weight. Furthermore, TAA treatment increased the liver to bodyweight ratio (%), while UR treatment has no effect on this index.

### 3.3. Effect of UR on Serum Liver Function and Histological Alterations

**Figure 2:**
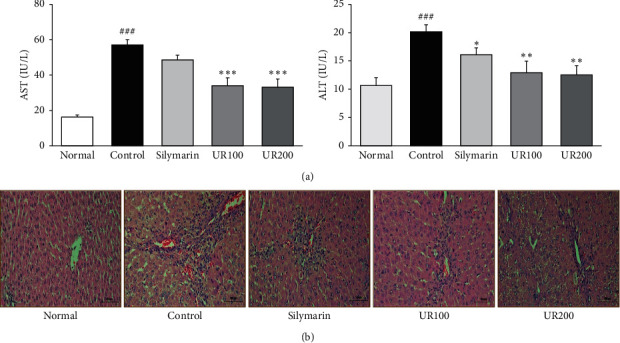
Effect of UR on serum liver function and histological alterations. (a) Effects of UR on serum levels of AST and ALT in rats. (b) Histological alterations in the liver. Liver tissue sections were stained with hematoxylin and eosin. Scale bar = 100 *μ*m (magnification, ×200). Data are expressed as the mean ± SEM (*n* = 7). Significance: ^####^*p* < 0.001 vs. the normal group and ^*∗*^*p* < 0.05, ^*∗∗*^*p* < 0.01, and ^*∗∗∗*^*p* < 0.001 vs. the ALI control group.

As shown in Figure 2(a), the serum levels of AST and ALT in the ALI control group were sharply increased by TAA injection. Their levels reached about 3.5-fold and-1.9 fold the levels in the ALI control group, respectively. Here, the marked elevation in liver enzyme activities induced TAA injection was significantly suppressed in the presence of UR. In addition, H&E staining (Figure 2(b)) indicated that liver tissue in the normal group showed normal architecture, whereas liver tissue in the ALI control group exhibited destruction of liver architecture, loss of cellular boundaries, inflammatory cell infiltration, and necrosis. Such characteristics were rehabilitated by UR treatment.

### 3.4. Effect of UR on Serum Ammonia and MPO Levels

**Figure 3:**
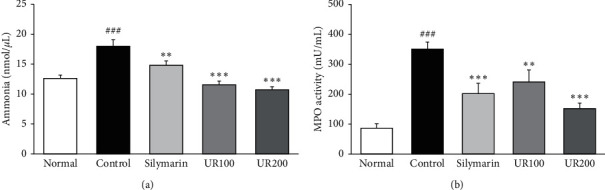
Effect of UR on serum ammonia and MPO levels. (a) Effects of UR on serum ammonia level in rats. (b) Effects of UR on serum MPO level in rats. Data are expressed as the mean ± SEM (*n* = 7). Significance: ^###^*p* < 0.001 vs. the normal group and ^*∗∗*^*p* < 0.01 and ^*∗∗∗*^*p* < 0.001 vs. the ALI control group.

As shown in Figure 3(a), rats receiving TAA dramatically increased the serum ammonia level compared with the normal group (12.61 ± 0.56 vs. 17.99 ± 1.09 nmol/*μ*L, *p* < 0.001). Serum ammonia levels were dose-dependently decreased in rats with UR treatment (11.57 ± 0.62 and 10.73 ± 0.51 nmol/*μ*L, respectively). Moreover, serum MPO sharply elevated 4.1-fold compared with the normal group (85.9 ± 14.9 vs. 350.9 ± 22.8 mU/mL, *p* < 0.001). Whereas, serum MPO levels were dose-dependently reduced in rats with UR treatment (241.1 ± 39.7 and 151.9 ± 18.3 mU/mL, respectively), as shown in Figure 3(b).

### 3.5. Effect of UR on Antioxidant Proteins

**Figure 4:**
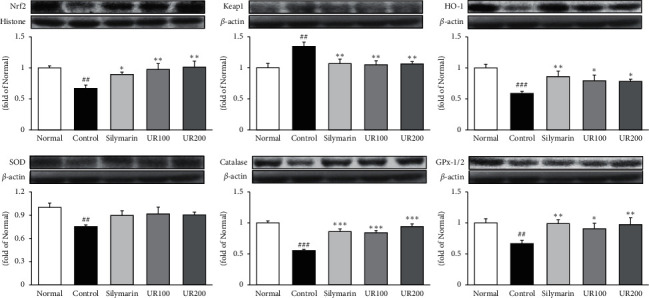
Effect of UR on antioxidant proteins. Effects of UR on the expression levels of Nrf2, Keap1, HO-1, SOD, catalase, and GPx-1/2 by the Western blotting assay. Data are expressed as the mean ± SEM (*n* = 7). Significance: ^##^*p* < 0.01 and ^###^*p* < 0.001 vs. the normal group and ^*∗*^*p* < 0.05^*∗∗*^*p* < 0.01, and ^*∗∗∗*^*p* < 0.001 vs. the ALI control group.

As shown in Figure 4, TAA injection markedly reduced the protein levels of Nrf2, HO-1, SOD, catalase, and GPx-1/2 by 0.33-, 0.41-, 0.25-, 0.44-, and 0.33-fold compared with the normal group, whereas it increased the protein level of Keap1 by 0.34-fold. Here, UR supplementation significantly upregulated all factors except for SOD and also downregulated Keap1. The data showed that UR alleviated TAA-induced acute liver injury through regulating antioxidant enzymes.

### 3.6. Effect of UR on Inflammatory Proteins

**Figure 5:**
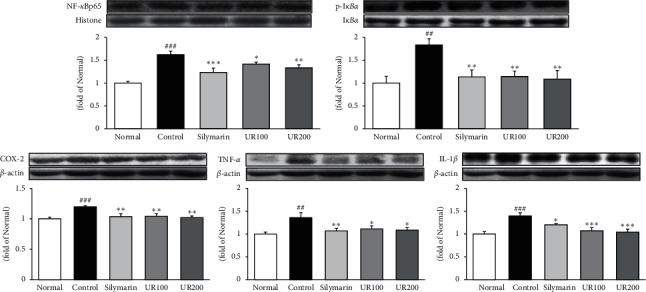
Effect of UR on inflammatory proteins. Effects of UR on the expression levels of p-I*κ*B*α*, NF-*κ*Bp65, COX-2, TNF-*α*, and IL-1*β* by the Western blotting assay. Data are expressed as the mean ± SEM (*n* = 7). Significance: ^##^*p* < 0.01 and ^###^*p* < 0.001 vs. the normal group and ^*∗*^*p* < 0.05, ^*∗∗*^*p* < 0.01, and ^*∗∗∗*^*p* < 0.001 vs. the ALI control group.

As shown in Figure 5, TAA injection obviously upregulated the protein levels of p-I*κ*B*α*, NF-*κ*Bp65, COX-2, TNF-*α*, and IL-1*β* by 0.83-, 0.62-, 0.2-, 0.36-, and 0.4-fold compared with the normal group. Here, UR supplementation significantly downregulated all factors. The data showed that UR inhibited TAA-induced acute liver injury through suppressing inflammation.

### 3.7. Effect of UR on Collagen Associated with Proteins

**Figure 6:**
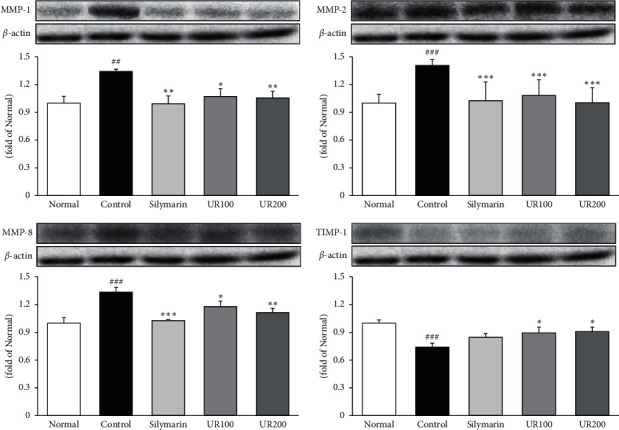
Effect of UR on collagen associated with proteins. Effects of UR on the expression levels of MMP-1, -2, and -8 and TIMP-1 by the Western blotting assay. Data are expressed as the mean ± SEM (*n* = 7). Significance: ^##^*p* < 0.01 and ^###^*p* < 0.001 vs. the normal group and ^*∗*^*p* < 0.05, ^*∗∗*^*p* < 0.01, and ^*∗∗∗*^*p* < 0.001 vs. the ALI control group.

As shown in Figure 6, cytokines increase metalloproteinases (MMPs) gene expression, and these enzymes degrade extracellular matrix (ECM), whereas TIMP-1 suppresses MMP-induced ECM degradation for the maintenance of intracellular homeostasis. Our results in this study revealed that UR significantly inhibited the expression of MMP-1, -2, and -8 (*p* < 0.01, *p* < 0.001, and *p* < 0.01, respectively); otherwise, it significantly elevated TIMP-1 (*p* < 0.05). The data suggested that UR improved TAA-induced acute liver injury through the inhibition of ECM degradation.

## 4. Discussion

Acute liver injury (ALI) is widely and extensively existed in our life, which can be caused by alcohol, drug abuse, food additives, viral infection, and radioactive damage. Ammonia is the waste produced by the nitrogen metabolism that is usually transported through the portal circulation to periportal hepatocytes. The urea cycle converts ammonia to urea in mammals. Urea is made in the liver and excreted in the form of urine by the kidneys [18]. Hyperammonemia is a life-threatening metabolic disturbance characterized by increased levels of toxic ammonia and can lead to severe neurologic emergency that can cause hepatic encephalopathy, liver cirrhosis, intracranial pressure crises, cerebral edema, and seizures [19]. The most common cause of hyperammonemia is acute liver injury/failure, chronic liver disease, and inherited metabolic diseases [20, 21].

*Uncaria rhynchophylla* (UR), a plant species used in various prescriptions of traditional Chinese medicine (TCM), has been used to extinguish wind, clear heat, arrest convulsions, and pacify the liver [22]. UR possesses alkaloids such as rhynchophylline and isorhynchophylline, and these alkaloids have been extensively used for the treatment of various diseases associated with the cardiovascular and central nervous systems [23]. Substantial experimental evidences indicate that rhynchophylline possesses pharmacological activities such as antihypertensive, antiarrhythmic, antianxiety, antiaddictive, anticonvulsant, sedative, and neuroprotective effects and also isorhynchophylline exerts anticancer and antimetastatic effects in hepatocellular carcinoma cells [24, 25].

Some toxic chemicals including thioacetamide (TAA), dimethylnitrosamine (DMN), carbon tetrachloride (CCl_4_), or acetaminophen can cause liver damage, which have been widely used to establish experimental animal models for evaluating the hepatoprotective activities of medicines [26, 27]. TAA, a selective hepatotoxin, is rapidly metabolized by cytochrome P450 to highly reactive metabolites causing inflammation, oxidative stress (OS), and hepatic necrosis [28]. Many studies have shown that hepatic inflammation usually occurs within few hours from TAA injection. Therefore, the correction between hepatic inflammation and OS in the course of liver damage is incontrovertible [29]. AST and ALT are substantially elevated when TAA-induced ALI. When hepatocytes are damaged, cell membrane permeability increases and AST and ALT, the essential enzymes in metabolic processes released into the blood [30]. UR significantly decreased AST and ALT levels and obviously improved the pathological damage of liver tissue. In the present work, the protective effects of UR against TAA-induced ALI in rats were investigated for the first time, and the results showed that UR markedly decreased serum AST and ALT levels; UR significantly improved TAA-induced ALI in rats, suggesting that UR may be one potential candidates for the treatment of ALI.

TAA can induce OS, inflammation, and lipid peroxidation to cause liver damage [31]. Notably, OS was reported to play an essential role in the pathogenesis of TAA-induced ALI. Therefore, traditional herbal medicines have been searched as strong candidate materials with antioxidant activity for alleviating the inflammatory disorder [32]. Myeloperoxidase (MPO) is known as an important mediator of the inflammatory response through the production of specific oxidative species, e.g., the hypohalous acids (HOCL) [33]. Serum MPO markedly elevated 4.1 fold compared with the normal group, whereas UR treatment led to a significant reduction dose-dependently in rats. The previous study reported that OS and inflammation are closely interrelated processes via secreting numerous nuclear factor-*κ*B- (NF-*κ*B-) mediated proinflammatory mediators [34]. NF-*κ*B is a key transcriptional regulator of the inflammatory response and regulates multiple functions such as immune responses and cell survival in Kupffer cells, hepatocytes, and hepatic stellate cells. Therefore, we performed the mechanistic study of UR based on OS and inflammation against TAA-induced ALI in the present study. In our work, UR markedly decreased the levels of TNF-*α*, IL-1*β*, and IL-6 though inhibiting NF-*κ*B activation in rats, suggesting that the anti-inflammatory effect may be one potential mechanism against TAA-induced ALI [35]. Furthermore, COX-2 and iNOS, an inducible type factor, can be rapidly promoted induced by NF-*κ*B induction in liver damage [36]. In the present study, UR suppressed TAA-induced inflammation via downregulating the protein expression levels of COX-2 and iNOS.

Moreover, we examined the expression of matrix metalloproteinase- (MMP-) 1, 2, and 8, and the tissue inhibitor of metalloproteinase- (TIMP-) 1. The extracellular matrix (ECM) is composed of collagen (95%), proteoglycans (aggrecans), elastin, laminins, and fibronectin [37]. MMPs are the primary enzymes implicated in ECM degradation, and irregular ECM turnover is involved in a variety of liver diseases [10, 38]. Meanwhile, active MMPs are mainly regulated by TIMPs, the chief MMP regulator. Here, TIMP-1 can directly promote the pericellular proteolysis of a great number of matrix and cell surface proteins or indirectly promote ECM deposition [39]. In this study, the ALI control group expressed increased MMP-1, -2, and -8 levels, whereas UR supplementation significantly blocked elevated protein levels. Otherwise, UR administration significantly elevated TIMP-1 compared with the ALI control group. These results suggest that the anticatabolic effect of UR may be due to the inhibition of MMP-1, -2, and -8.

## 5. Conclusions

**Figure 7:**
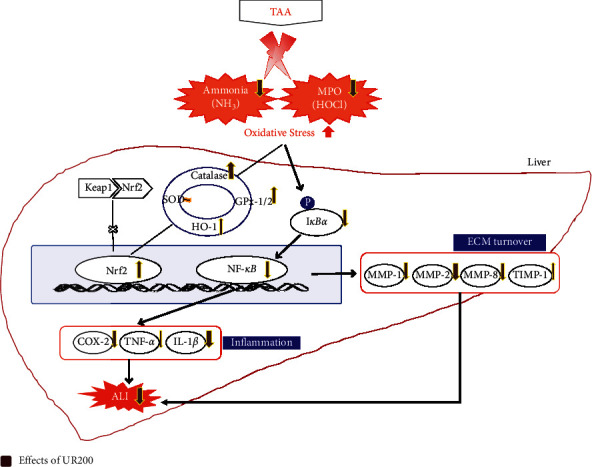
Predicted mechanisms in liver tissue on UR200 administration.

In conclusion, UR showed hepatoprotective effects against TAA-induced ALI in rats via suppressing both hepatic oxidative stress and inflammation by NF-*κ*B activation, as shown in Figure 7. This study suggests that UR may be developed as an effective healthcare product for liver protection.

## Figures and Tables

**Table 1:** The effect of UR on the liver weight, bodyweight, and LW/BW ratio.

Group	Liver weight (g)	Bodyweight (g)	LW/BW ratio (%)
Normal	8.5 ± 0.16	238.6 ± 2.24	3.6 ± 0.07
TAA-treated mice			
Control	10.1 ± 0.27^###^	198.5 ± 4.16^###^	5.1 ± 0.12^###^
Silymarin	9.5 ± 0.16	198.4 ± 2.26	4.8 ± 0.09^*∗*^
UR100	9.8 ± 0.32	202.9 ± 3.64	4.8 ± 0.10
UR200	9.5 ± 0.16	200.3 ± 3.89	4.7 ± 0.06

Control, distilled water-administered and TAA-induced ALI rats. Silymarin, silymarin 100 mg/kg bodyweight administered and TAA-induced ALI rats; UR100, UR 100 mg/kg bodyweight administered and TAA-induced ALI rats; UR200, UR 200 mg/kg bodyweight-administered and TAA-induced ALI rats. Data are the mean ± SEM, *n* = 7. Significance, ^###^*P* < 0.001 versus the normal group and ^*∗*^*P* < 0.05 versus the ALI control group.

## Data Availability

The datasets used and analyzed during this study are available from the corresponding author upon request.
